# Atypical ubiquitin E3 ligase complex Skp1-Pam-Fbxo45 controls the core epithelial-to-mesenchymal transition-inducing transcription factors

**DOI:** 10.18632/oncotarget.2825

**Published:** 2014-11-25

**Authors:** Ming Xu, Changhong Zhu, Xian Zhao, Cheng Chen, Hailong Zhang, Haihua Yuan, Rong Deng, Jinzhuo Dou, Yanli Wang, Jian Huang, Qin Chen, Bin Jiang, Jianxiu Yu

**Affiliations:** ^1^ Institute of Oncology & Department of Oncology, Shanghai 9th People's Hospital, Shanghai Jiao Tong University School of Medicine, Shanghai, China; ^2^ Department of Biochemistry and Molecular Cell Biology & Shanghai Key Laboratory of Tumor Microenvironment and Inflammation, Shanghai Jiao Tong University School of Medicine, Shanghai, China; ^3^ State Key Laboratory of Oncogenes and Related Genes, Shanghai Jiao Tong University School of Medicine, Shanghai, China

**Keywords:** Epithelial-mesenchymal transition (EMT), EMT-Inducing Transcription Factors (EMT-TFs), Fbxo45, miR-27a*, ubiquitin degradation

## Abstract

Epithelial-mesenchymal transition (EMT) plays a critical role in the development of tumor metastases by enhancing migration/invasion. One of the hallmarks of EMT is loss of E-cadherin and gain of N-cadherin expression, which are regulated by the core EMT-inducing transcription factors (EMT-TFs), such as Zeb1/2, Snai1/2 and Twist1. Here, we find that EMT-TFs can be dynamically degraded by an atypical ubiquitin E3 ligase complex Skp1-Pam-Fbxo45 (SPF^Fbxo45^) through the ubiquitin proteasome system (UPS). The key step is recognition of EMT-TFs by Fbxo45 through its SPRY domain for Zeb2, or F-box domain for the other three EMT-TFs Snai1, Snai2 and Twist1, respectively. The K48-linkaged ubiquitination capability on Zeb2 relies on its functional SBD domain. In addition, miR-27a* can directly down-regulate the expression of Fbxo45, preventing degradation of EMT-TFs and thus ensuring EMT phenotype. We suggest that Fbxo45 is a key node of the miR-27a*/Fbxo45/EMT-TFs signaling axis.

## INTRODUCTION

The epithelial-mesenchymal transition (EMT) is first recognized as a feature in the embryonic process [1], which is essential for numerous developmental processes including mesoderm and neural tube formation [1-3]. EMT has also been shown to occur in wound healing [4], organ fibrosis [5, 6] and the initiation of metastasis for cancer progression, by which epithelial cells lose their cell polarity and cell-cell adhesion to gain enhanced migratory and invasive properties [6-8]. A variety of extracellular stimuli have the potential to induce EMT, including components of the extracellular matrix (ECM), as well as soluble growth factors, such as tumor growth factor beta (TGF-β), epithelial growth factor (EGF) and fibroblast growth factor (FGF) [6]. Growing evidences uncover that EMT-inducing transcription factors (EMT-TFs) directly or indirectly play critical roles in the embryogenesis and cancer initiation/progression, and also highlight the existence of significant overlap among EMT-TFs in their regulatory signals, target genes and mechanisms of action [9-11]. Members of the SNAIL family of transcriptional regulators, termed Snai1 and Snai2 (*also named* Slug), have emerged as a key node during the cell regulatory networks to trigger the occurrence of EMT event [8, 12-14], in which one of the hallmarks is loss of E-cadherin and/or gain of N-cadherin expression [15, 16], thereby resulting in disruption of cell junctions and desmosomes [17]. The transcriptional factor Twist can induce EMT by repressing E-cadherin *via* binding to its promoter [18], and also through affecting other EMT-TFs, such as induction of Snai1 [19] and Snai2 [20]. The zinc finger transcription factors Zeb1 and Zeb2 (*also named* SIP1), downstream of the Snail and Twist families in the EMT interactome, also make a pivotal contribution to this regulation in the initiation of metastasis for cancer progression [21-23].

At the transcriptional regulatory level, EMT-TFs can be either regulated by transcription factors of themselves or others through binding to their promoter regions [24], or suppressed by microRNAs through the 3′-terminal untranslated region (UTR) for further mRNA degradation or blocking the translational action of gene expression [25-27]. More importantly, however, the ubiquitin proteasome system (UPS) which recruits E1, E2 and E3 ubiquitin enzymes precisely regulating the degradation of these transcription factors that control cell cycles and regulate gene expressions in response to diverse stress including oxidative stress, growth factors or interferon stimuli [28-30]. E3 ubiquitin-protein ligases are critical in the UPS, as they allow transfer of activated ubiquitin from E2 enzymes to the target protein and mediate the specificity of substrate recognition [31]. The Skp, Cullin and F-box containing complex (*i.e.*, SCF complex) is a multisubunit E3 ubiquitin ligase catalyzing the ubiquitination of proteins destined for proteasomal degradation [32]. Canonical SCF complex is composed of invariable core components (Cul1, Skp1 and RBX1) and a variable F-box protein module, which specifically recruits substrates [33, 34]. Approximately 70 F-box proteins have been identified in mammals, nevertheless most remain to be matched to specific substrates [31]. Exceptionally, Fbxo45, a member of the F-box protein family, can not form the canonial SCF complex due to the point mutation (E42R) in the F-box functional domain resulting in it being incompatible with the scaffold protein Cul1 [35]. Instead, Fbxo45 molecule as a component of Skp1-Pam-Fbxo45 complex, namely SPF^Fbxo45^, plays a part in the embryogenesis, synapse formation or apoptosis in tumor [35-37].

Here, we have identified that the atypical E3 ligase SPF^Fbxo45^ mediates the degradation of EMT-TFs through the ubiquitin-proteasome pathway, which directly affects the EMT processes in mammalian cancer cells. We also find that miR-27a* transcriptionally suppresses the expression of Fbxo45 to influence the formation of SPF^Fbxo45^ complex, resulting in stabilization of EMT-TFs which contribute the EMT initiation and cancer progression.

## RESULTS

### Expression Levels of EMT-TFs in Tumor or Cancer Cell Lines

**Figure 1:**
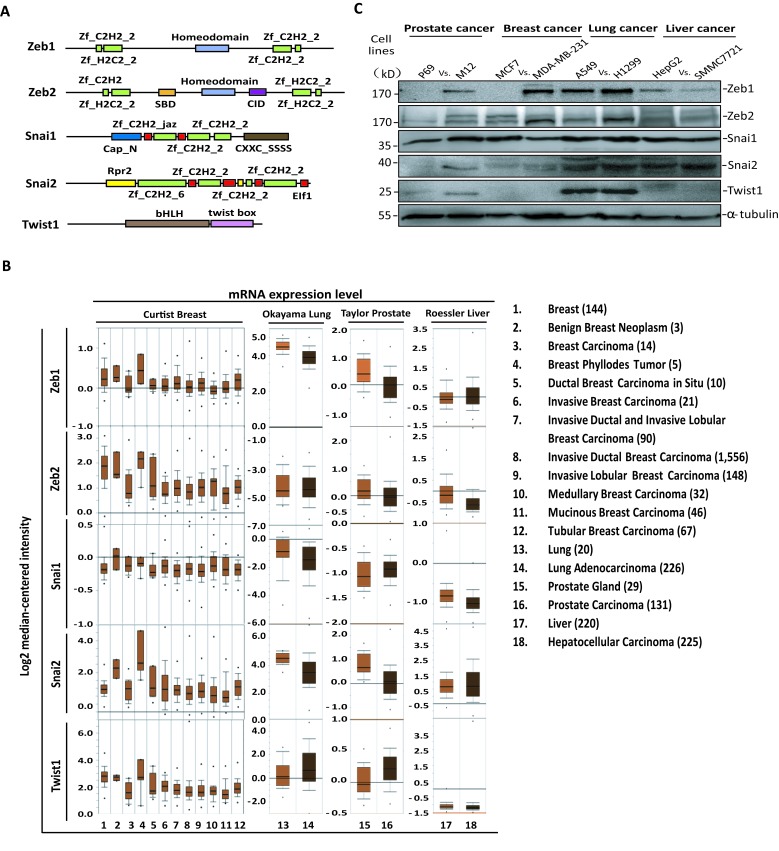
Expression levels of EMT-TFs in tumors and cancer cell lines A. Structures of Zeb1/2, Snai1/2 and Twist1. B. mRNA levels of EMT-TFs were derived from the oncomine database, including Curtis Breast (2136 cases), Okayama Lung (246 cases), Taylor Prostate (160 cases) and Roessler Liver (445 cases), which contain normal tissues and benign or malignant tumor samples. Box-and-whisker plots show the upper and lower quartiles (25-75%) with a line at the median, whiskers extend from the 10th to the 90th percentile, and dots correspond to minimal and maximal values. C. The protein levels of EMT-TFs were compared between carcinoma cell lines and non- or less-malignant tumor cell lines by using Western-blot analysis. The soft agar colony transformation or migration assay was referred to distinguish malignant degree of the experimental cancer cell lines (data not shown). Malignant degree: prostate cancer cell lines, P69 < M12; breast cancer cell lines, MCF7 < MD-MBA-231; lung cancer cell lines, A549 < H1299; liver cancer cell lines, HepG2 < SMMC7721. ‘kD’ (kilo-dalton) means the molecular weight of protein.

There are some similarities on the protein structures of core EMT-TFs, including Zeb1, Zeb2, Snai1, Snai2 and Twist1. They possess multiple H_2_C_2_ or C_2_H_2_ zinc finger domains whereas Twist1 contains only the helix-loop-helix (HLH) domain, which are all responsible for binding to the specific DNA motif to regulate transcription activities for further downstream signaling or cellular actions (Fig 1A). It is necessary to understand the regulatory mechanisms of these transcription factors, or their effects on the cellular behaviors under specific conditions, particularly during the EMT-inducing progression in tumor development.

To investigate the mRNA expression levels of EMT-TFs in tumor, the data from the oncomine databases including Curtis Breast (2136 cases), Okayama Lung (246 cases), Taylor Prostate (160 cases) and Roessler Liver (445 cases) were collected for analysis, showing that the mRNA levels in carcinoma samples were not obviously increased when compared to those of normal tissue or benign neoplasm; on the contrary, they were decreased to some extent (Fig 1B). However, the protein levels of EMT-TFs are usually much higher in the clinical tumor samples than those in the normal tissues according to the previous reports [38, 39]. Furthermore, we found the protein levels of these EMT-TFs were significantly enhanced in the carcinoma cell lines compared to those in the non-malignant tumor cell lines (Fig 1C). To rule out this was caused by any potential effect on translation, the half-lives of Zeb1 and Zeb2 were selectively measured. Their half-lives in the carcinoma cell line H1299 were longer than those in the non-malignant tumor cell lines A549, supporting the concept that EMT-TFs (Zeb1/2) are more stable in the carcinoma cell lines (Supplementary Fig S1). Above data indicate that these transcription factors controlling tumor cell EMT processes may more rely on the protein stabilities other than the mRNA regulations.

### Fbxo45, as a Module of SPF^Fbxo45^ Complex, Induces the Degradation of EMT-TFs Including Zeb1, Zeb2, Snai1, Snai2 and Twist1

The stability of Snai1 has been confirmed to be mediated through ubiquitin-proteasome system [40]. In *Xenopus laevis*, the protein stabilities of Twist1, Snai1, Snai2 and even Zeb2 are associated with the F-box protein partner of paired (Ppa), a component of E3 ubiquitin enzyme, which functions on the embryonic or neural crest development [30, 41]. Thus, we postulated the atypical E3 ubiquitin ligase SPF^Fbxo45^ complex might also make contributions to EMT processes by mediating the ubiquitin-dependent degradation of EMT-TFs, particularly Zeb1 and Zeb2 proteins in cancer cells.

**Figure 2:**
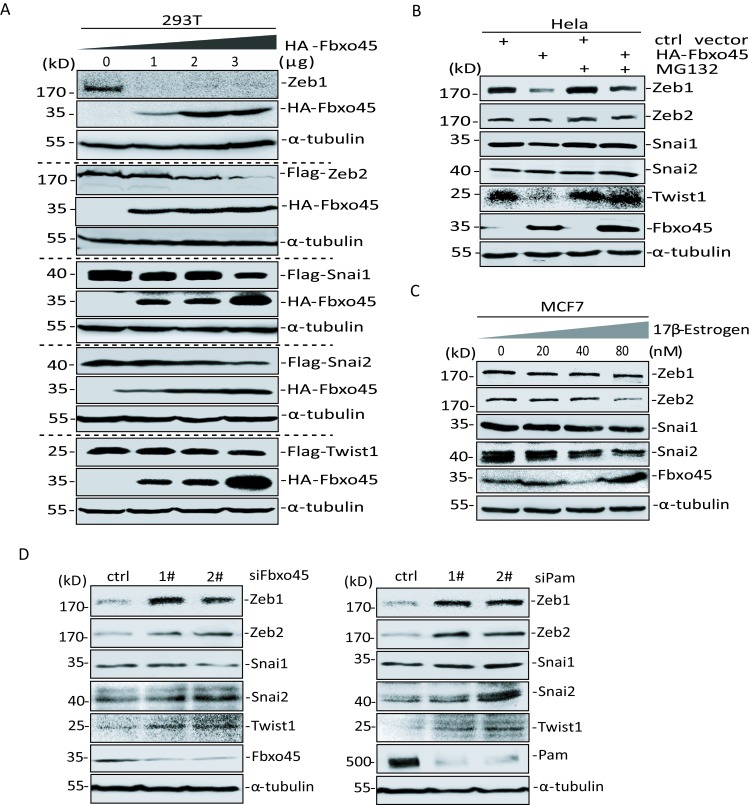
Fbxo45 induces degradation of core EMT-TFs A. Immunoblot for core EMT-TFs in 293T cells transfected Zeb1, Zeb2, Snai1, Snai2 or Twist1 with increasing amount of Fbxo45. B. Immunoblot for detecting endogenous core EMT-TFs in HeLa cells transfected with or without myc-tagged Fbxo45, and harvested after treatment with 20μM of MG132 for 4 hours. C. Immunoblot for endogenous Zeb1, Zeb2, Snai1 and Snai2 in MCF7 cells treated with indicated different concentrations of 17β-estrogen for 24 h in the phenol-free medium to induce the expression of endogenous Fbxo45. D. Immunoblot for endogenous core EMT-TFs in HeLa transfected with siRNAs for Fbxo45 or Pam.

To test whether the degradation of these core EMT-TFs is involved in Fbxo45, we expressed Fbxo45 together with Zeb1, Zeb2, Snai1, Snai2 and Twist1 in HEK293T cells, respectively, and found that the protein levels of these five exogenous EMT-TFs were gradually decreased by increasing the amount of Fbxo45 (Fig 2A). These five endogenous EMT-TFs were also down-regulated by ectopically expressing Fbxo45 in HeLa cells; however, further treatments with a specific proteasome inhibitor MG132 blocked the degradation of EMT-TFs induced by Fbxo45 (Fig 2B). Moreover, the protein levels of endogenous Zeb1, Zeb2, Snai1 and Snai2 were obviously decreased under the treatment of 17β-estrogen for 24 hours, by which endogenous Fbxo45 was induced at high expression levels in MCF7 cells (Fig 2C), which was consistent with as reported before that Fbxo45 is an estrogen-induced gene owing to its promoter region containing transcriptional activation sites of estrogen receptor (ER) [42]. We failed to detect the Twist1 protein due to an extremely low expression level in MCF7 cells. In contrast, the EMT-TFs were stabilized, especially Zeb1, Zeb2, Snai2 and Twist1, when using siRNAs for knockdown of either Fbxo45 or Pam, which each module is one of SPF^Fbxo45^ complex executing the function of ubiquitin E3 (Fig 2D). Taken together, these results indicate that core EMT-TFs including Zeb1, Zeb2, Snai1, Snai2 and Twist1 are potentially the specific substrates of the atypical E3 ligase complex SPF^Fbxo45^, which mediates their degradation thereby controlling the occurrence of tumor cell EMT processes and even tumor metastasis.

### Functional Domains of Fbxo45 for Ubiquitination on Zeb2

**Figure 3:**
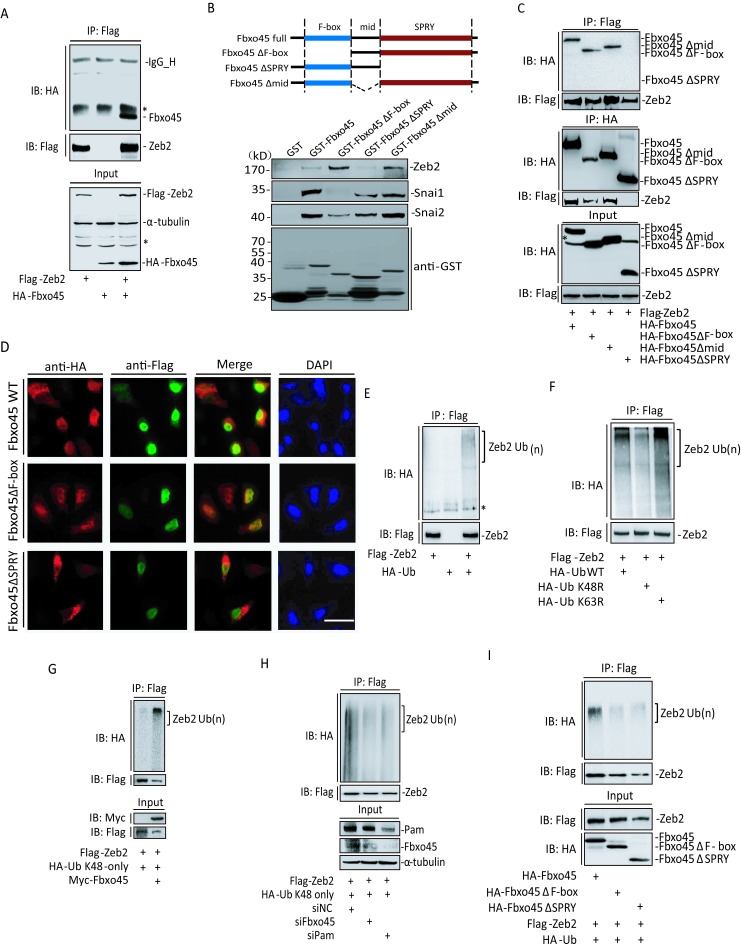
Functional domains of Fbxo45 for ubiquitination of Zeb2 A. Lysates from 293T cells transfected Flag-Zeb2 with or without HA-Fbxo45 were immunoprecipitated (IP) with anti-Flag M2 antibody, and then immunoblotted. Total lysates were also used as Input for immunoblotting analysis. B. Bacterially expressed GST or GST–Fbxo45, -Fbxo45ΔF-box, -Fbxo45Δmid, or -Fbxo45ΔSPRY fusion proteins and Glutathione-Sepharose beads were incubated with lysates of HeLa cells transfected with Zeb2, Snai1 or Snai2, respectively. The proteins associated with GST–tagged Fbxo45 forms, bound on the Glutathione-Sepharose beads were washed five times with the RIPA buffer before immunoblotting. C. Lysates from 293T cells transfected Flag-Zeb2 with HA-Fbxo45, -Fbxo45Δmid, -Fbxo45ΔF-box or -Fbxo45ΔSPRY were immunoprecipitated with anti-Flag or anti-HA antibodies, and immunoprecipitates were resolved by SDS-PAGE for Western-blot analysis. D. U2OS cells transfected with Flag-Zeb2 and HA-Fbxo45, -Fbxo45ΔF-box or -Fbxo45ΔSPRY were stained using the primary antibodies of anti-Flag M2 or anti-HA, and the second antibodies of Alexa Fluor 568 anti-mouse or Alexa Fluor 488 anti-Rabbit, respectively. Scale: 25μm. E-I. Zeb2 ubiquitination assays by using IP expriments under different conditions: Flag-Zeb2 with or without HA-Ub (E); Flag-Zeb2 with HA-Ub WT, K48R or K63R (F); Flag-Zeb2 and HA-Ub K48-only with or without myc-Fbox45 (G); Flag-Zeb2 and HA-Ub K48-only with siRNA control, siRNAs for Fbxo45 or siRNAs for Pam (H); Flag-Zeb2 and HA-Ub with HA-Fbxo45, -Fbxo45ΔF-box or -Fbxo45ΔSPRY (I).

Next, we wanted to investigate the biochemical mechanism by which SPF^Fbxo45^ mediates the ubiquitination on Zeb2, or other EMT-TFs. Since SPF^Fbxo45^ complex is an ubiquitin E3 ligase, its function in recognizing substrates mostly depends on the Fbxo45 module. Consistent with this notion, exogenously expressed Fbxo45 efficiently co-immunoprecipitated with Zeb2 (Fig 3A and Supplementary Fig S2), as well as Zeb1, Snail, Snai2 and Twist1 (Supplementary Fig S2), but another F-box protein Fbxo9 could not efficiently bind with Zeb2 (Supplementary Fig S3). To exclude out indirect interaction between Fbxo45 and EMT-TFs, GST-tagged wide-type (WT) or truncated forms of Fbxo45 were expressed and purified from bacteria DE3 *E.Coli* for further GST-pull down experiments, showing that Fbxo45 could directly bind to endogenous Zeb2, Snai1 and Snai2 in HeLa cells (Fig 3B), or to exogenous expressed Snai1, Snai2 and Twist1 in HEK293T cells (Supplementary Fig S4). More interestingly, Fbxo45 recognized substrates for further ubiquitination probably *via* utilizing different functional domains, *i.e.*, SPRY for the recognition of Zeb2; F-box for the recognition of Snai1/2, Twist1 (Fig 3B and Supplementary Fig S4). In addition, ectopically expressed Zeb2 was co-immunoprecipitated with WT or truncated forms of Fbxo45, and these results also confirmed that the SPRY domain was essential for Fbxo45 binding to Zeb2 protein (Fig 3C), which was in line with the GST-pull down experiments (Fig 3B). Moreover, microscopy immunofluorescence studies revealed co-localization of wide-type Fbxo45 or its deletion form Fbxo45ΔF-box in nuclear with Zeb2, but the form of Fbxo45ΔSPRY preferred to remain in the cytoplasm rather than in the nucleus (Fig 3D).

To further investigate whether the ubiquitination of EMT-TFs is truly mediated by SPF^Fbxo45^ complex, we used Zeb2 as a representative for in-depth studies. Indeed, the ubiquitination assays showed that Zeb2 protein was ubiquitinated *in vivo* in the presence of the proteasome inhibitor MG132 (Fig 3E). As known, the ubiquitin K48-linkages on target proteins for proteasomal degradation whereas the ubiquitin K63-linkages are required for cell signaling events in DNA damage response or cytokine signaling [43]. To determine what kind of lysine-linkage ubiquitination occurs on Zeb2 protein, we co-transfected Zeb2 with wild-type, mutant K48R or K63R ubiquitin, respectively. As shown in Fig 3F, IP-Western blotting results showed the poly-ubiquitin chains on Zeb2 mostly relied on K48-linkages rather than K63-linkages for degradation. To further confirm this, ubiquitin K48-only (all other lysines K6, K11, K27, K29, K33, K63 are mutated into Arginines) was used to show that Zeb2 was indeed ubiquitinated by K48-linkages, which was enhanced by overexpressed Fbxo45 (Fig 3G). In addition, we found that Fbxo45 much more effectively promoted the ubiquitination of Zeb2 when compared with Fbxo9 (Supplementary Figure S5)

As expected, knockdown of either Fbxo45 or Pam would disrupt the formation of SPF^Fbxo45^ complex as an ubiquitin E3 ligase. Indeed, the immunoprecipitation experiments showed ubiquitination on Zeb2 was significantly impaired when either Fbxo45 or Pam was down-regulated by siRNAs (Fig 3H). Compared to WT Fbxo45, the truncated forms of Fbxo45ΔF-box and Fbxo45ΔSPRY distinctly lost the ability of ubiquitination on Zeb2 (Fig 3I), being in line with the knockdown experiments (Fig 3H). Taken together, SPF^Fbxo45^ complex is a specific ubiquitin E3 ligase of Zeb2, even the other EMT-TFs, and Fbxo45 module is responsible for recognizing diverse substrates *via* its SPRY or F-box domain.

### SBD Domain of Zeb2 is Essential for its Ubiquitination

**Figure 4:**
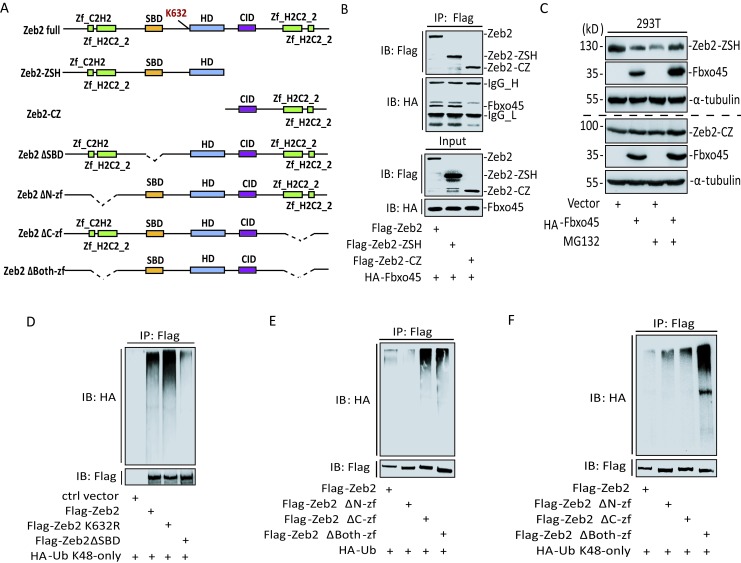
SBD domain of Zeb2 is essential for its ubiquitination A. Deletion /truncation strategy of Zeb2. B. Lysates from 293T cells transfected HA-Fbxo45 with Flag-Zeb2, -Zeb2-ZSH or -Zeb2-CZ were immunoprecipitated (IP) with anti-Flag M2 antibody, and then immunoblotted. Total lysates were also used as Input for immunoblotting analysis. C. 293T cells were transfected with Flag-Zeb2-ZSH (three upper panels) or -Zeb2-CZ (three lower panels) with or without HA-Fbxo45. Lysates harvested after treatment with DMSO or MG132 (20μM) for 4h were analyzed by Western-blotting. D. Lysates from 293T cells transfected HA-Ub K48-only with Flag-Zeb2, -Zeb2 K632R or -Zeb2ΔSBD were immunoprecipitated with anti-Flag M2 antibody, and then immunoblotted. E-F. Lysates from 293T cells transfected HA-Ub (E) or HA-Ub K48-only (F) with Flag-Zeb2, -Zeb2ΔN-zf, -Zeb2ΔC-zf or -Zeb2ΔBoth-zf were immunoprecipitated with anti-Flag M2 antibody, and then immunoblotted.

Since Zeb2 protein can be degraded by SPF^Fbxo45^ complex through the ubiquitin pathway, it is necessary to determine what domain or region on Zeb2 protein is responsible for binding with Fbxo45 for its ubiquitination. To this end, we generated a series of truncated forms of Zeb2 for further experiments (Fig 4A). First, we found that Fbxo45 could be easily detected by co-immunoprecipitation with Zeb2-ZSH but not with Zeb2-CZ (Fig 4B). In good agreement with this, co-expression of Fbxo45 could decrease only Zeb2-ZSH but not Zeb2-CZ (Fig 4C, lanes 1, 2). Moreover, MG132 treatment prevented the degradation of Zeb2-ZSH mediated by Fbxo45 while had no significant effects on the protein levels of Zeb2-CZ with or without Fbxo45 expression (Fig 4C, lanes 3, 4). More interestingly, we found that the K48-linkage ubiquitination on Zeb2 were obviously impaired when the SBD domain was deleted (Fig 4D, lane 4), while SBD deletion did not affecting the interaction between Fbxo45 and Zeb2 (Supplementary Fig S6). K632, which is located at the N-terminal of HD domain, has been reported as a potential ubiquitination site on Zeb2 on the basis of mass spectrum identification [44]. Thus, we wanted to validate whether K632 on Zeb2 protein is covalently modified with the K48-linkage ubiquitination, but unfortunately, the mutation of K632R had no decreased effect on ubiquitination (Fig 4D, lane 3) and the degradation rate of Zeb2 induced by Fbxo45 (Fig 4D, lane 3 and Supplementary Fig S7).

Due to the structural similarity of EMT-TFs except Twist1, which contain multiple C_2_H_2_ or H_2_C_2_ zinc finger domains (Fig 4A), we identified that both N-terminal and C-terminal zinc finger domains did not affect the interaction of Zeb2 with Fbxo45 (Supplementary Fig S8); On the contrary, the zinc finger domain deletion forms including Zeb2ΔN-zf, Zeb2ΔC-zf and Zeb2Δboth-zf increased ubiquitinations on Zeb2 protein, whatever the K48 or other lysine-linkages (Fig 4E-F). Taken together, SBD domain of Zeb2 was essential for its ubiquitination mediated by Fbxo45.

### Fbxo45 is a Direct Target of miR-27a* that Mediates EMT Processes

**Figure 5:**
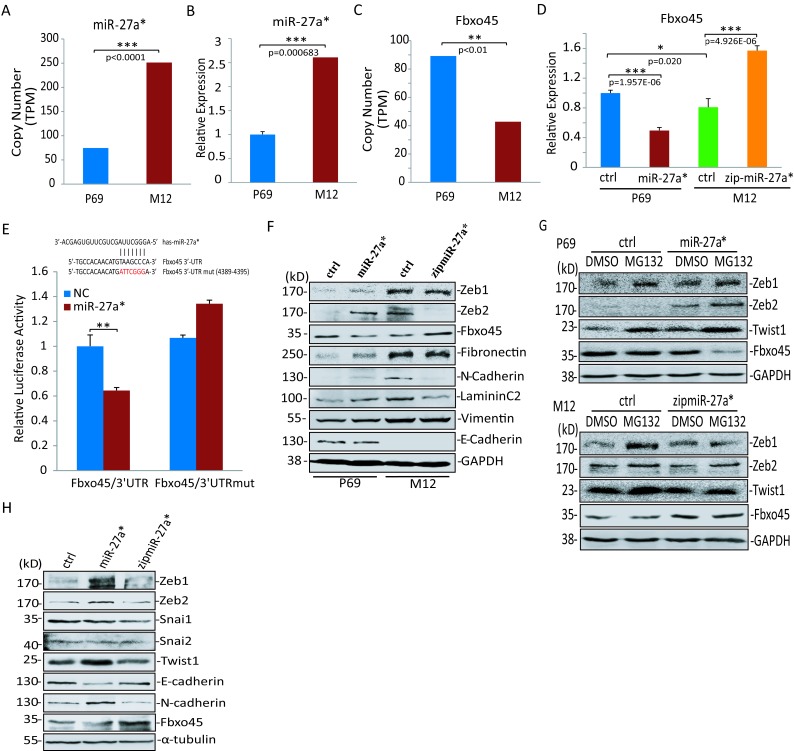
Fbxo45 is a direct target of miR-27a* that mediates EMT processes A, C. The expression levels of miR-27a* (A) and Fbxo45 mRNA (C) in P69 and M12 were shown according to the data of miRNA transcriptome sequencing and DGE sequencing, respectively [46]. TPM means transcripts per million. B. Real-time PCR in ΔΔCt methods were used to confirm the expression levels of miR-27a* in P69 and M12. One representative experiment out of three was shown. D. Fbxo45 mRNA levels in stable cell lines miR-27a*-overexpressing P69 and miR-27a*-silencing M12 (zipmiR-27a*). P69 or M12 stably infected with the same lentiviral empty vector was used as a control (ctrl). E. Fbxo45 3′-UTR wide-type or mutant form at the position where is complementary to the 5′ seed region of miR-27a* was subcloned into psiCheck2 vector. 293T cells were transfected with psiCheck2 containing WT or mutant Fbxo45 3′-UTR and miR-27a* or non-specific control vector. The Renilla luciferase activity was normalized on the constitutive activity of firefly luciferase. Data are the mean±S.E.M. of three independent experiments. F-G. Immunoblot for Fbxo45, core EMT-TFs (Zeb1, Zeb2, Twist1) and their associated EMT markers (Fibronectin, N-cadherin, Laminin C2, Vimentin and E-cadherin) in stable cell lines P69-miR-27a* or M12-zipmiR-27a* (F); Immunoblot for detection of protein stability of core EMT-TFs under the treatment with 20μM of MG132 for 4 h before harvested (G). GAPDH is for a loading control. H. Immunblot for Fbxo45, N-cadherin, E-cadherin and five core EMT-TFs in stable cell lines PC3-ctrl, PC3-miR-27a* and PC3-zipmiR-27a*. α-tubulin is for a loading control.

P69 is a non-tumorigenic, non-metastatic prostate epithelial cell line while M12 is a highly tumorigenic and metastatic subline derived from P69 [45, 46]. The progression of these two cell lines from rarely tumorigenic to fully metastatic provides an experimentally accessible tool for the study of the metastatic phenotype acquisition. We sequenced the miRNA transcriptomes and Digital Gene Expression Profilings (DGEs) [47] of two sublines P69 and M12, respectively, by using the Illumina high-throughput sequencing technology, and noted that a special miRNA, miR-27a* in M12 was much higher than that in P69 (Fig 5A), which was confirmed by real-time qRT-PCR (Fig 5B). Conversely, endogenous Fbxo45 mRNA levels in the metastatic subline M12 were lower than that in P69 (Fig 5C). Based on above data, we speculated that miR-27a* might repress Fbxo45 to disrupt SPF^Fbxo45^ complex thereby affecting on the downstream biological processes. To identify the regulatory role of miR-27a*, P69 or M12 was infected with the lentiviral vector system for stable expressing miR-27a*(Supplementary Fig S9) or zipmiR-27a*, respectively. As shown in Fig 5D, Fbxo45 mRNA was significantly repressed by overexpression of miR-27a* in P69 whereas it was increased by zipping of miR-27a* in M12. According to the prediction by miRanda-mirSVR, miR-27a* has a perfect targeting site in the 3′UTR of Fbxo45 gene, indicating that Fbxo45 is a predicted target gene of miR-27a*. To test whether miR-27a* can directly target Fbxo45, luciferase reporter assays were carried out in HEK293T cells. The wide-type Fbxo45 3′UTR or mutant with the 7-bp sequence complementary to the 5′ seed region of miR-27a* (Fbxo45 3′UTR^mut^) was subcloned downstream of the reporter Renilla luciferase gene of psiCheck2 vector (Fig 5E). The reporter constructs were transfected into cells along with miR-27a* mimics, as expected, miR-27a* dramatically reduced the wide-type reporter activity, whereas the mutant reporter was not affected (Fig 5E). In consistent with the mRNA expressions, the protein levels of Fbxo45 were also negatively regulated by over-expressing or blocking miR-27a* (Fig 5F). Collectively, Fbxo45 is a direct target of miR-27a* in cells.

Since SPF^Fbxo45^ complex as an ubiquitin E3 ligase mediates the degradation of EMT-TFs, however it is still unclear how the enzymatic activity is suppressed for stabilizing EMT-TFs to promote tumor cell metastasis processes. To investigate whether miR-27a* can stabilize EMT-TFs by repressing Fbxo45, we extracted lysates of stable cell lines P69-miR-27a* and M12-zipmiR-27a* for Western blotting analysis, respectively. The results showed over-expression of miR-27a* could indeed increase the protein levels of Zeb1 or Zeb2 in P69, whereas inhibition of miR-27a* in M12 reduced the protein levels of EMT-TFs, especially Zeb2 (Fig 5F). Moreover, Western blot analysis showed that the protein stabilities of EMT-TFs in P69 or M12 were associated with the effect of proteolysis, and miR-27a* delayed degradation through repressing Fbxo45 thereby affecting the E3 enzyme activity of SPF^Fbxo45^ complex (Fig 5F-G). In contrast to epithelial cells, mesenchymal cells can migrate easily resulting from their losing the expression of E-cadherin and highly expressing those of N-cadherin, fibronectin and vimentin. Consistent with this notion, the expressions of N-cadherin, Laminin C2 and Fibronectin were upregulated by ectopic expression of miR-27a* in P69 and downregulated by zipping miR-27a* in M12 (Fig 5F), although the expression levels of E-cadherein and Vimentin appeared not to be affected by miR-27a*. Similar roles of miR-27a* were also observed in PC3 cell line (Fig 5H). Taken together, these data demonstrated that miR-27a* mediates tumor cell EMT processes through stabilizing the core EMT-TFs by direct suppression of Fbxo45 that is a module of SPF ubiquitin E3 complex.

### miR-27a*/Fbxo45/EMT-TFs Axis Controls EMT Initiation and Cancer Progression

**Figure 6:**
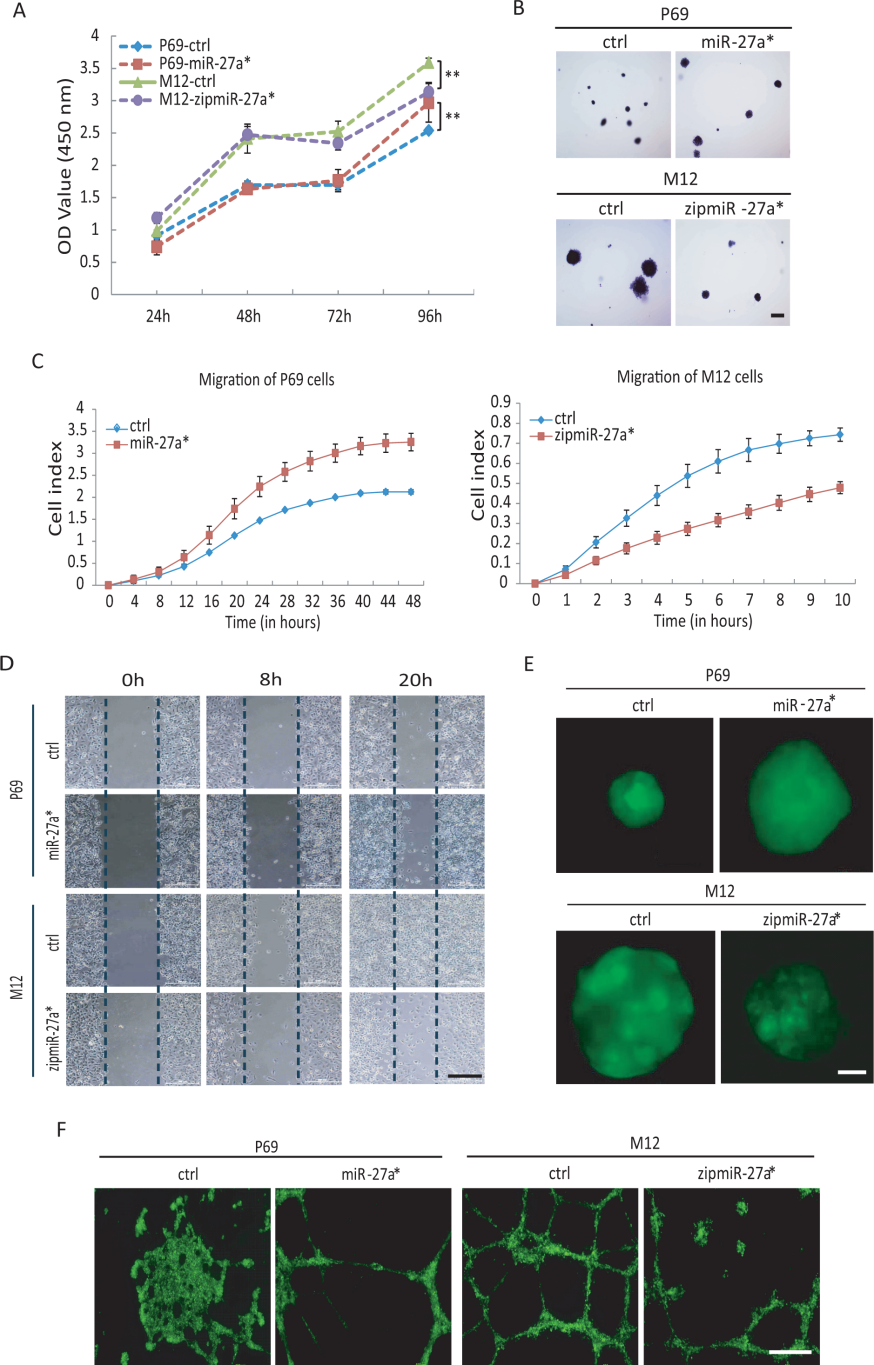
miR-27a*/Fbxo45/EMT-TFs axis controls EMT initiation and cancer progression Four stable cell lines P69-ctrl, P69-miR-27a*, M12-ctrl and M12-zipmiR-27a* were used in the following experiments, A. CCK-8 proliferation assay at time course of 24, 48, 72, 96 h. B. Soft agar colony formation assays. The photos were taken at 14 days. Scale: 50μm. C. RTCA-migration assays by using the x CELLigence RTCA-DP system. D. Wound healing assays for migration. Scale: 200 μm. E. 3D cell culture assays. The photos were taken at 10 days. Scale: 20 μm. F. Vasculogenic mimicry assays. The photographs were taken with microscope 20 hours later. Scale: 500 μm.

To investigate the roles of miR-27a* in inducing the occurrence of EMT, a series of phenotypes were investigated. As shown in Fig 6A, over-expression of miR-27a* significantly increased cell proliferation of P69 after culturing for 96 h while blocking miR-27a* slowed down cell proliferation of M12, indicating that miR-27a* had potential abilities to promote the cell growth. Moreover, the soft agar colony-forming assays were conducted to test whether miR-27a* affects the transforming potential of each stable P69/M12 cell lines. As expected, compared to the control vector transfected cells, P69 cells transfected with miR-27a* showed promotion of colony growth while M12 cells transfected with zipmiR-27a* suppressed anchorage-independent growth (Fig 6B). Cells undergo a complete EMT includes losing their cell-cell adhesion and gaining solitary migration characteristics [48]. In line with those properties, miR-27a* significantly promoted cell migration in P69 while expressed zipmiR-27a* suppressed the forceful migratory ability of M12 cells by using Real-Time Cell Analysis [46, 49] (Fig 6C) and wound healing assays as a complementary approach (Fig 6D). In addition, 3D cell-culture assays also showed ectopical expression of miR-27a* promoted P69 cells to grow much bigger spheroids, whereas inhibition of miR-27a* restrained the sizes of spheroids in M12 cells (Fig 6E).

The assays of vasculogenic mimicry (VM) on matrix-gel can illustrate the interrelationships between the genetically dysregulated invasive tumor cell, the microenvironment and the malignant switch [50], and VM formation can be used to assess the degree of malignancy. Thus, we used the VM assays to confirm the roles of miR-27a* in controlling the invasiveness abilities of cancer cells [49]. Consistently with above results, over-expression of miR-27a* in P69 strongly induced formation of vascular-like shape while blocking miR-27a* in M12 reduced (Fig 6F). Laminins, a family of extracellular matrix glycoproteins, are the major noncollagenous constituent of basement membranes. They have been implicated in a wide variety of biological processes including cell adhesion, differentiation, migration, signaling, neurite outgrowth, metastasis and vasculogenesis [51, 52]. In agreement with that, Lamini C2 was obviously regulated by miR-27a* in both cell lines (Fig 5E), thereby directly affecting VM formation (Fig 6F).

**Figure 7:**
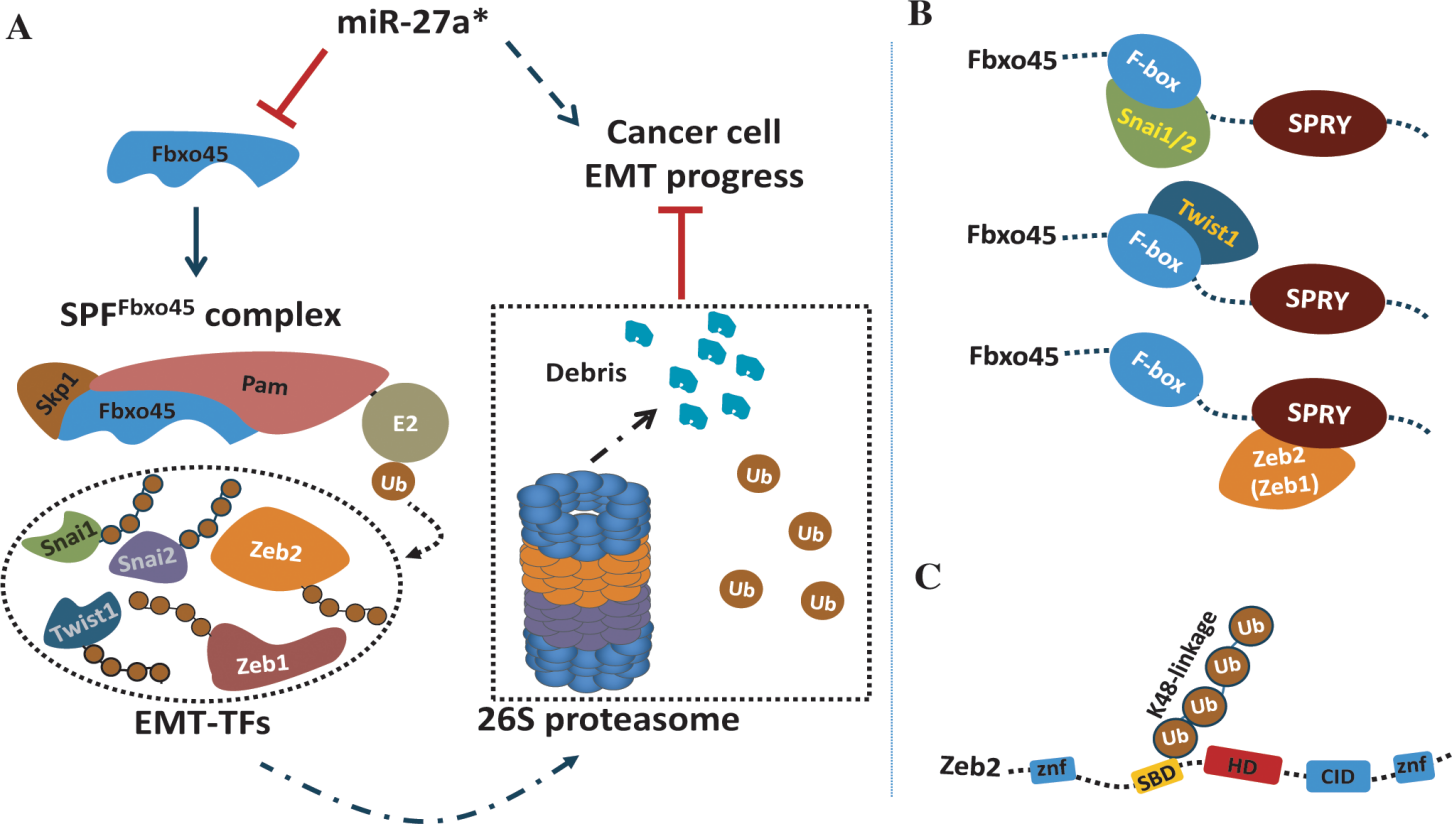
miR-27a*/Fbxo45/EMT-TFs axis controls EMT in tumor cells A. An atypical E3 ubiquitin ligase SPF^Fbxo45^ directly mediates the degradation of core EMT-TFs including Zeb1/2, Snai1/2 and Twist1 by the ubiquitin proteasome pathway, which maintains epithelial characteristics of cells. However, high level of miR-27a* promotes the occurrence of epithelial-to-mesenchymal transition (EMT) events whereby miR-27a* represses Fbxo45 by directly targeting 3′-UTR of Fbxo45 mRNA, thereby disrupting the formation of SPF^Fbxo45^ E3 complex, finally resulting in stabilization of those core EMT-TFs. B. The module Fbxo45 in SPF^Fbxo45^ complex can recognize diverse proteins by its SPRY or F-box domain to fulfill the further ubiquitination on the specific substrates. Deletion of either F-box or SPRY resulting in losing contact to Skp1 or Pam, respectively, impedes the E3 ligase function of SPF^Fbxo45^ complex. C. SBD is the main domain and essential for K48-linkage poly-ubiquitination on Zeb2 for further UPS degradation.

Finally, miR-27a*, as a potential onco-miR, can trigger tumor cell EMT initiation and promote cancer progression *via* directly targeting Fbxo45 to disrupt the SPF^Fbxo45^ complex, thereby maintaining the protein stabilities of EMT-TFs, along with characteristic phenotypes of malignant cells (Fig 7A).

## DISCUSSION

Epithelial-mesenchymal transition is the initial transition process of malignant transformation, but also the crucial step of cancer cell invasion and metastasis whereby losing of cell-cell adhesion ability to increase cell migration. In the past few decades, many scientists in the world have focused on the core EMT-TFs and their regulatory networks during cancer initiation and progression [11, 53], however, the underlying mechanisms have not been fully elucidated. We analyzed abundant mRNA array or deep sequencing data from clinical tumor tissue samples including breast cancer, lung cancer, prostate cancer and liver cancer, and found that the mRNA levels of EMT-TFs including Zeb1, Zeb2, Snai1, Snai2 as well as Twist1 in tumor samples were not significantly altered compared to those in normal tissue or benign neoplasm, which were mismatched with the high protein levels of EMT-TFs in malignant tumors [18, 54, 55] or cancer cell lines (Fig 1B-C). These implied a novel mechanism underlying higher protein stabilities of EMT-TFs during cancer cell EMT initiation and progression.

Our data indicated these EMT-TFs are labile proteins that are quickly degraded by an atypical E3 ligase of SCF, SPF^Fbxo45^ complex, through the ubiquitin-proteasome system. In SPF^Fbxo45^ complex, Pam (also called MycBP2 in mammals) contains a typical RING-finger structure at its COOH-terminal, which is responsible for the E2-Ubiquitin recognition and transferring ubiquitin molecular to specific substrates [35]. Moreover, Fbxo45 as a substrate binding module interacts directly or indirectly with Pam through its SPRY domain, and with Skp1 *via* its NH2-terminal F-box domain, respectively [35] (Fig 7B). Either exogenous Fbxo45 or estrogen-stimulated endogenous Fbxo45 enhanced the enzyme activity of SPF^Fbxo45^ for ubiquitin-dependent degradation of those EMT-TFs. Consistently, ablation of either Pam or Fbxo45 stabilized EMT-TFs through reducing ubiquitination on substrates, such as Zeb2, which strongly implied that the SPF^Fbxo45^ complex was the specific ubiquitin E3 ligase of EMT-TFs. We also demonstrate that Fbxo45 could recognize diverse substrates (*e.g*., Zeb2) mainly depends on its SPRY domain, which is in line with the previous reports [37, 56]. Likewise, F-box domain which is usually linked to the adapter protein Skp1 [57, 58], can be as well as in another mode for substrate recognition thereby assisting the ubiquitination of SPF^Fbxo45^ on specific substrates (Fig 7B). In addition, SPRY domain is essential for Fbxo45 nuclear localization or interaction with intranuclear substrates, at least with Zeb2, to mediate the further degradation.

Zeb2 as a key transcription factor inducing EMT occurrence, plays a vital role in a variety of tumor cells [23, 59-61]. In malignant tumor cells, Zeb2 and even other EMT-TFs are more stable, thus up-regulating the downstream oncogenic signals and triggering EMT and promoting cancer initiation, progression and development. The mechanism underlying high protein stability of Zeb2 in cancer cells, however, is still unclear. A previous study shows in the process of EMT, Snai1 can promote a transcription of a natural antisense transcript of Zeb2, to prevent the slicing of introns containing in the Zeb2 5′-UTR region where IRES (internal ribosome entry site) recruits the ribosome to translate the mRNA to raise the Zeb2 protein expression [62]. In this study, we demonstrate a novel molecular mechanism is responsible for the regulation of protein stability of EMT-TFs including Zeb2 in cells. Zeb2 was used as a representative for studying on its ubiquitination and functional domains associated with the orchestration of Fbxo45 module of SPF^Fbxo45^. Our data demonstrate that the protein level of Zeb2 is strictly controlled by the SPF^Fbxo45^complex and its degradation relies on the formation of K48-linkage poly-ubiquitin chains. Deletions of either F-box or SPRY domain of Fbxo45 significantly weakened the ubiquitination on Zeb2, suggesting that Zeb2 protein degradation is connected with the correct formation of SPF^Fbxo45^-Zeb2 complex. On the other hand, Zeb2 protein comprises multiple functional domains, including the DNA binding domain (Zinc Finger, Zf), CtBP protein interaction domain (CID), homeodomain (HD) and Smad protein interaction domain (SBD). Our results suggest that Zeb2 interacts with Fbxo45 by the N-terminal region Zeb2-ZSH other than the C-terminal region Zeb2-CZ. Moreover, SBD is essential for K48-linkage poly-ubiquitination on Zeb2, probably due to its containing precise lysines for the ubiquitination (Fig 7C).

In recent years microRNAs have also been reported in regulation of EMT occurrence of tumor cells. In this study, we also demonstrate that miR-27a* directly targeted Fbxo45 thus enhancing the protein stabilities of EMT-TFs to promote tumor cell EMT processes by using P69 and M12 cell lines, which are an ideal pair of cell model for EMT study. Combining with previous results on global mRNA deep sequencing results of P69 and M12 cell lines, we showed that miR-27a* significantly controlled EMT phenotype of tumor cells, such as cell proliferation, migration and tumor transformation. In conclusion, we propose that a novel signaling axis miR-27a*/Fbxo45/EMT-TFs may play a crucial role in EMT occurrence, cancer initiation and metastasis. Therefore, our findings may have important clinical implications, as microRNA or E3 enzymes are potential targets, which repressing miR-27a* or enhancing the activity of E3 ubiquitin ligase SPF^Fbxo45^ represents a new therapeutic target for ideal treatment of cancer or even other diseases.

## MATERIALS AND METHODS

### Antibodies

Rabbit anti-Laminin C2 (SC-20776), HA-probe (SC-57592), goat anti-Snai2 (SC-10437) and mouse anti-N-Cadherin (SC-8424) were from Santa Cruz Biotechnology. Rabbit antibodies against Pam (ab86078), Fbxo45 (ab126521), GAPDH (ab37168), and mouse anti-Twist1 (ab50581) were from Abcam. Rabbit anti-E-cadherin (#3195), -Zeb1 (#3396), and mouse anti-Myc (#2276) were from Cell signaling technology. Rabbit anti-Zeb2 (NBP1-77179) was from Novus. α-tubulin (#66031-1-1g) antibody was from Proteintechnology. Anti-Flag M2 (F1804), -HA (MMS-101P) and -Vimentin V9 (V6630) mouse antibodies were from Sigma-Aldrich. Snai1 (A52437) rabbit antibodies were from ABclonal. Rabbit anti-fibronectin (GTX112794) was from GeneTex. Protein G Plus/A agarose suspension (#IP05) was purchased from Calbiochem.

### Construction of Plasmids

The human full-length Zeb1 and Zeb2 tagged with 3xFlag were cloned into pCDH-CMV-MCS-EF1-GreenPuro vector (System Bioscences) for exogenous expression. Flag-tagged Snai1, Snai2 and Twist1 were stored in our lab. Full-length Fbxo45 and Fbxo9 cDNA were inserted into pCMV-HA, pCMV-Myc or pGEX-4T-1 vectors. HA-Fbxo45 and 3xFlag-Zeb2 mutants were generated by mutagenesis PCR using KOD Hot Start DNA polymerase (TOYOBO). The synthetic DNA oligos (Sangon), miR-27a* (forward and reverse) or zipmiR-27a* (forward and reverse) were used to generate an mRNA transcripts from pGreen-puro vector (System Bioscences) for further lentivirus packaging in HEK293FT cells with pCMV-dR8 and pMD.2G (System Bioscences). To generate psiCheck2-Fbxo45 3′UTR, 810-bp fragment containing the complementary DNA sequence with miR-27a* seed region (4389-4395bp) were cloned between sites XhoI and NotI of the psiCheck2 vector (Promega). Further we mutated the recognition site of miR-27a* by mutagenesis PCR using the following primers Fbxo45 3′UTR^mut^ forward and reverse. All plasmids were checked carefully by sequencing the entire insert. Oligo nucleotide sequences are provided in the supplementary Table-1.

### Cell Culture and Transfection

HEK293T, HeLa, U2OS, HepG2, PC3, MCF7 and MDA-MB-231 cells were maintained at 37°C and 5% CO_2_ in DMEM supplemented with 10% fetal bovine serum (FBS), and penicillin/streptomycin at 100U/ml. P69 and M12 were cultured in RPMI-1640 (Hyclone) supplemented with 5% fetal bovine serum (FBS) (Hyclone), 10ng/ml EGF, 0.2μM Dexamethasone, 5μg/ml Insulin, 5μg/ml Transferrin, 5μg/ml Sodium Selenite, 50μg/ml Gentamicin, 2.5μg/ml Fungizone. A549, H1299 and SMMC7721 cells were cultured in RPMI-1640 with 10% FBS, and penicillin/streptomycin at 100U/ml. MCF7 cells were also cultured in DMEM with 10% FBS, but without phenol for the 17β-estrogen inducing experiments. P69 or M12 cells stably expressing miR-27a* or zipmiR-27a*, respectively, were in addition supplemented with 20μg/ml puromycin. Plasmid transfection was performed using Lipofectamine 2000 (Invitrogen) according to manufacturer's instructions.

### GST Protein Pull-down Assays

The pGEX-4T-1 plasmid (GE healthcare) was used to clone pGEX-Fbxo45 and its truncated forms. All plasmids were induced to express insert genes in DE3 *E.coli* bacteria (Sangon) with 0.5 mM IPTG at 16°C for 10 hrs. Pellets were dissociated on ice to clear in bacteria protein extraction regent (Thermo Fisher) containing 100 μg/ml lysozyme (Sigma), 12.5 μl/ml protease inhibitor cocktails with EDTA (Sangon), 2 U/ml DNase I (Thermo Fisher). Glutathione sepharose 4B (GE healthcare) were used to purify the GST-tagged protein and eluted in sequential buffer 1 (50mM Tris-HCl (pH8.0), 20mM Glutathion), buffer 2 (50mM Tris-HCl (pH8.0), 10mM Glutathion) and concentrated for further pull down experiments. Endogenous Zeb1/2, Snai1/2, Twist1 from HeLa cell extraction in RIPA buffer (50 mM Tris-HCl (pH7.4), 150 mM NaCl, 0.5% NP-40, and complete protease inhibitor cocktail tablet (Roche) were incubated and rotated with purified GST-tagged proteins and Glutathione sepharose 4B beads at 4°C overnight. RIPA buffer washed precipitations were resolved by SDS-PAGE for western-blot detection using antibodies.

### Immunoprecipitation / Western Blotting

Immunoprecipitation (IP) and Co-Immunoprecipitation (Co-IP) were performed with 300 to 500 μg of HEK293T lysates prepared using modified RIPA buffer (50 mM Tris-HCl (pH7.4), 150 mM NaCl, 0.5% NP-40 for Co-IP or 1% NP-40 for IP, and complete protease inhibitor cocktail tablet (Roche). Exogenous Flag-tagged proteins (Zeb1/2, Snai1/2, Twist1) were precleared with protein A/G-agarose beads (Santa Cruz Biotechnology) prior to immunoprecipitation with mouse anti-Flag M2 antibody. Immunocomplexes bound to agarose beads were washed four times in the same RIPA buffer. Western blotting was performed by resolving whole-cell lysates or immune-complex by SDS-PAGE and blotting onto Immobilon-P membranes (Millipore Corporation). The supersensitive ECL kit (Millipore Corporation) was used and the blotting was developed with LASmini4000 (General Electric Co) as described before[63].

### Immunofluorescence

U2OS cells were co-transfected with 3xFlag-Zeb2 (GFP fragment in the plasmid was truncated by mutation methods) and HA-tagged wide-type or truncated forms of Fbxo45 on 12mm coverslips prior treatments. After 24 hours, cells were fixed in 4% paraformaldehyde/PBS at room temperature for 20 minutes, permeabilized in 0.2% Triton X-100/PBS for 5 minutes, blocked in 10% goat serum in PBS for 1 hr. First antibodies diluted in 5% goat serum in PBS (rabbit anti-HA 1:250 and mouse anti-Flag 1:500) were incubated at 4°C overnight. Further, cells were washed 3 times with PBS and stained with secondary antibodies diluted in 2% goat serum in PBS (Fluor 488 anti-Rabbit 1:500 and Fluor 568 anti-mouse 1:500) for 1hr at room temperature in the dark. Cells were washed one time and then incubated with 0.1μg/ml DAPI for 5 minutes. Covered slides were observed and taken photography under Nikon microscope.

### Dual Luciferase Reporter Assays

The dual luciferase reporter assays were performed as described before[46, 64]. The psiCheck2 vector (Promega) was used to clone the 3′-UTR or mutant form of human Fbxo45. Renilla luciferase activity is then used to assess the effect of the 3′-UTR on transcript stability and translation efficiency. Firefly luciferase is used to normalize transfections and eliminates the need to transfect a second vector control. 293T cells were seeded in the wells of a 24-well plate 1 day pre-transfection and then each well transfected with a mixture of 200 ng 3′-UTR luciferase reporter vectors and 400 ng miR-27a* mimics (Sangon), scrambled microRNA as a negative control. Twenty-four hours posttransfection, cells were lysed and luciferase activity was measured using a NOVOstar (BMG Labtech) and the Dual-Luciferase Reporter Assay System (Promega). The ratios of Renilla luciferase to firefly luciferase were calculated to generate the columns.

### Soft Agar Colony Assay

The soft agar assay for colony formation was performed as described before[49, 64]. Briefly, 1.2% Agar in Milli-Q water was autoclaved and cool to 40°C in a water bath. Using warmed 2x RPMI-1640 with 4% FBS and antibiotics to mix with equal volume of the agar solution to give 0.6% Agar in 1x RPMI with 2% FBS. 2ml of mixture was transferred into 6-well plates and allow at least 30 minutes for temperature to solidify as the bottom agar gel. The same procedure was performed to prepare 0.35% agar gel containing P69 or M12 cells (1500 cells/well) infected with lentivirus expressing miR-27a* or zipmiR-27a*, respectively. Put the 6-Well plates in 4°C for 1-2hrs, and then cover the 1x RPMI-1640 medium on the top of agar gel. Incubate plates at 37°C in humidified incubator for 10 to 30 days. Stain plates with 0.5 ml of 0.005% Crystal Violet for more than 1 hour and count colonies using a dissecting microscope.

### Three-dimensional (3D) Cell Culture

The 3D cell culture assay was performed as described before[46]. Briefly, 5 μl of MatriX gel (Millipore) and 5μl of cell solution (1-2×10^4^ cells/ml) were mixed and transferred into the 8-well slides (IBIDI) and covered with complete cell culture medium after the gel being solidified. The slides were incubated at 37°C and 5% CO_2_ cell incubator for 6 to 10 days depending on the cell types, then for observation under microscope.

### Vasculogenic Mimicry

The vasculogenic mimicry experiment was performed as described before [49].

### Migration Assay by Wound Healing

This method for analysis of migration was conducted as described previously[49].

### Migration by Real-Time CellAnalysis (RTCA)

Cell migration experiments were performed using the xCELLigence RTCA-DP system (Roche, Mannheim, Germany) as described previously [49, 63-64].

### Statistical Analysis

Experiments were performed at least three times, and representative results were shown. Statistical analysis of qRT-PCR or luciferase assay was performed with a two-tailed unpaired *t*-test with triplicate or quadruplicate sets. p<0.05 was considered statistically significant.

## SUPPLEMENTARY MATERIAL AND FIGURES


