# Correction: Atypical ubiquitin E3 ligase complex Skp1-Pam-Fbxo45 controls the core epithelial-to-mesenchymal transition-inducing transcription factors

**DOI:** 10.18632/oncotarget.27253

**Published:** 2019-10-08

**Authors:** Ming Xu, Changhong Zhu, Xian Zhao, Cheng Chen, Hailong Zhang, Haihua Yuan, Rong Deng, Jinzhuo Dou, Yanli Wang, Jian Huang, Qin Chen, Bin Jiang, Jianxiu Yu

**Affiliations:** ^1^ Institute of Oncology & Department of Oncology, Shanghai 9th People’s Hospital, Shanghai Jiao Tong University School of Medicine, Shanghai, China; ^2^ Department of Biochemistry and Molecular Cell Biology & Shanghai Key Laboratory of Tumor Microenvironment and Inflammation, Shanghai Jiao Tong University School of Medicine, Shanghai, China; ^3^ State Key Laboratory of Oncogenes and Related Genes, Shanghai Jiao Tong University School of Medicine, Shanghai, China

**This article has been corrected:** During the manuscript editing, the α-tubulin band in Figure 2B was accidentally misplaced. The corrected Figure 2B is shown below. The authors declare that this correction does not change the results or conclusions of this article.

Original article: Oncotarget. 2015; 6:979–994. 979-994. https://doi.org/10.18632/oncotarget.2825

**Figure 2:** Fbxo45 induces degradation of core EMT-TFs. (**A**) Immunoblot for core EMT-TFs in 293T cells transfected Zeb1, Zeb2, Snai1, Snai2 or Twist1 with increasing amount of Fbxo45. (**B**) Immunoblot for detecting endogenous core EMT-TFs in HeLa cells transfected with or without myc-tagged Fbxo45, and harvested after treatment with 20μM of MG132 for 4 hours. (**C**) Immunoblot for endogenous Zeb1, Zeb2, Snai1 and Snai2 in MCF7 cells treated with indicated different concentrations of 17β-estrogen for 24 h in the phenol-free medium to induce the expression of endogenous Fbxo45. (**D**) Immunoblot for endogenous core EMT-TFs in HeLa transfected with siRNAs for Fbxo45 or Pam.



