# Supplementary material for: Atypical ubiquitin E3 ligase complex Skp1-Pam-Fbxo45 controls the core epithelial-to-mesenchymal transition-inducing transcription factors
Source: Oncotarget. 2014 Nov 25;6(2):979–94. doi: 10.18632/oncotarget.2825 (PMC4359269; doi:10.18632/oncotarget.2825)
Supplement: Supplementary file 1 [file oncotarget-06-979-s001.pdf]

Atypical ubiquitin E3 ligase complex Skp1-Pam-Fbxo45 controls the core epithelial-to-mesenchymal transition-inducing transcription factors

Supplemental Figure Legends

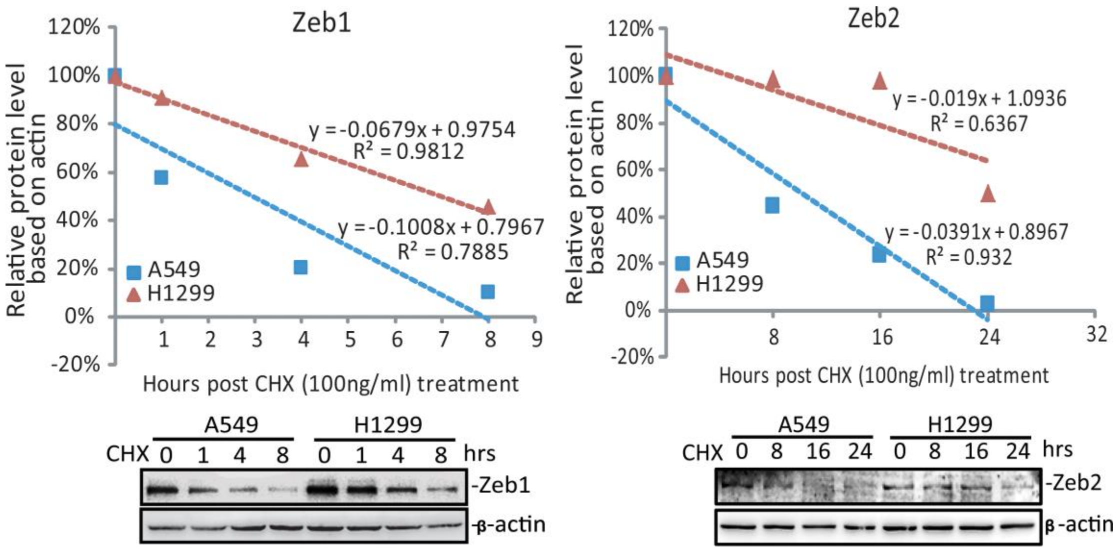

Figure S1: Half-lives of endogenous Zeb1 and Zeb2 in A549 and H1299 cells. Cells were treated with cycloheximide (100 μg/ml) and harvested in SDS lysis buffer at the indicated time points. Relative amounts of Zeb1 and Zeb2 were quantified by using ImageJ software and normalized to **β**-actin, and plotted (the experiment repeated three times).

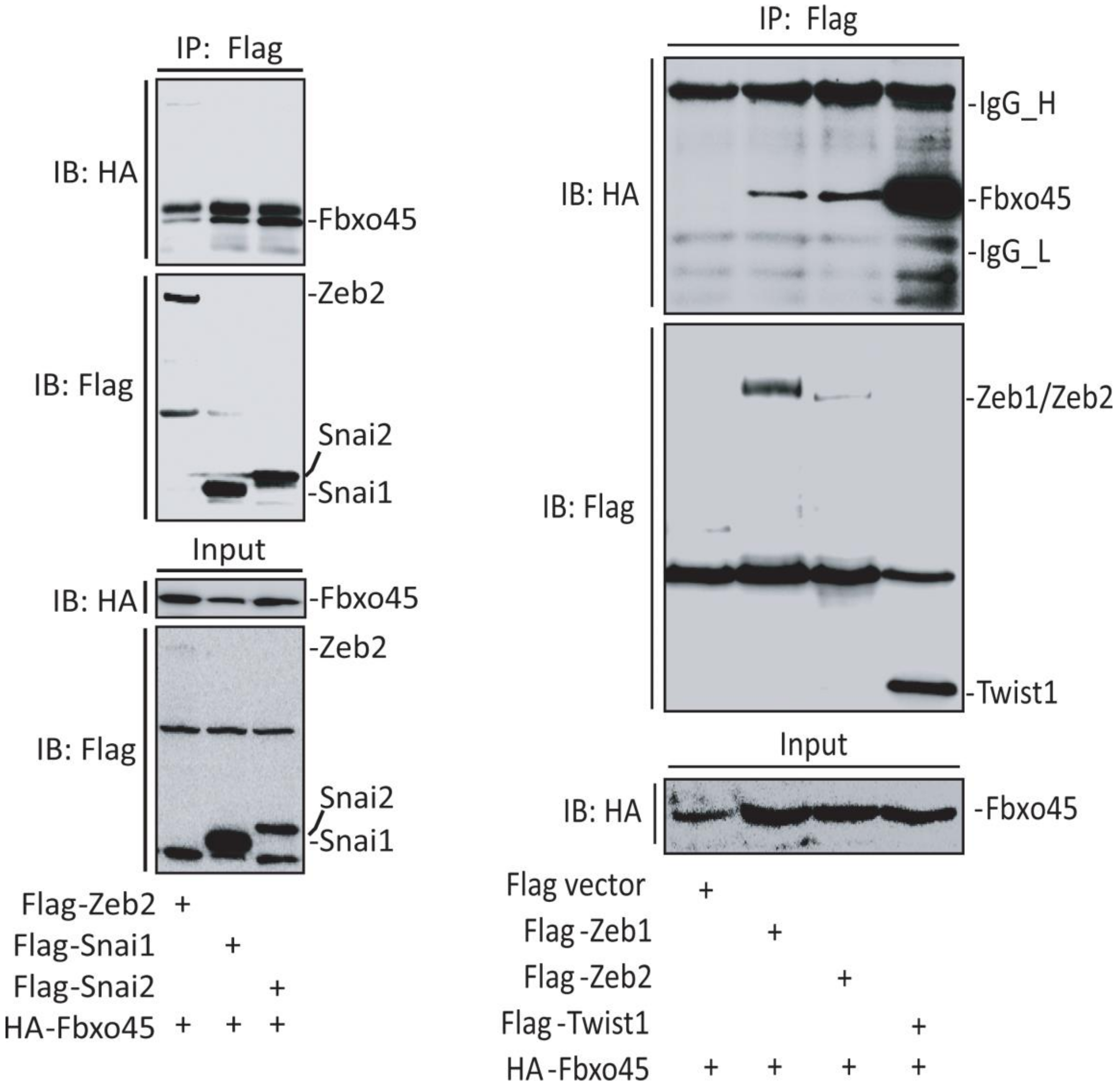

Figure S2: Functional domains of Fbxo45 for ubiquitination on Zeb2. Zeb1/2, Snai1/2, Twist1 with Flag-tag were immunoprecipitaed from lysates of HEK293T cells using the Flag M2 antibody, and immunoprecipitates were resolved by SDS-PAGE and Fbxo45 protein bands were detected by HA-probe antibody using western-blot method.

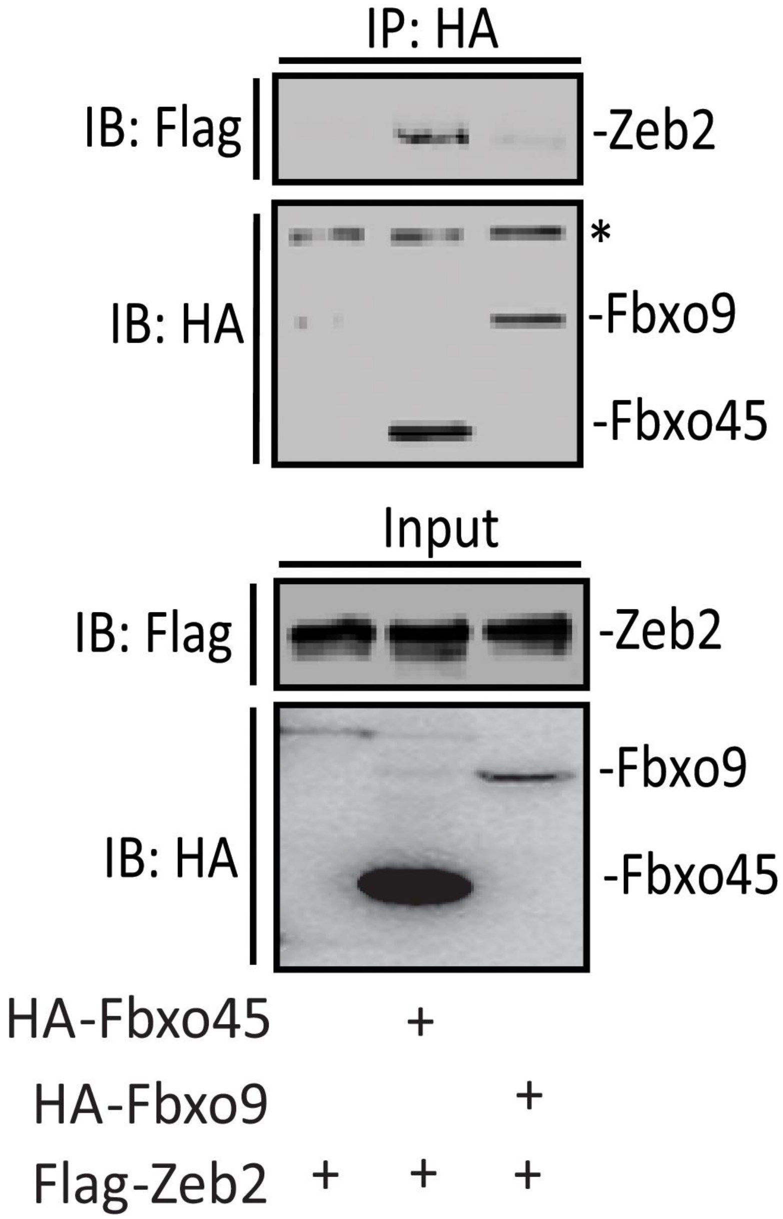

Figure S3: HA-tagged Fbxo45 or Fbxo9 were immunoprecipitaed with anti-HA antibody from lysates of HEK293T cells transfected, and immunoprecipitates were resolved by SDS-PAGE and Zeb2 protein bands were detected by anti-Flag M2 antibody. Asterisk indicated the non-specific bands.

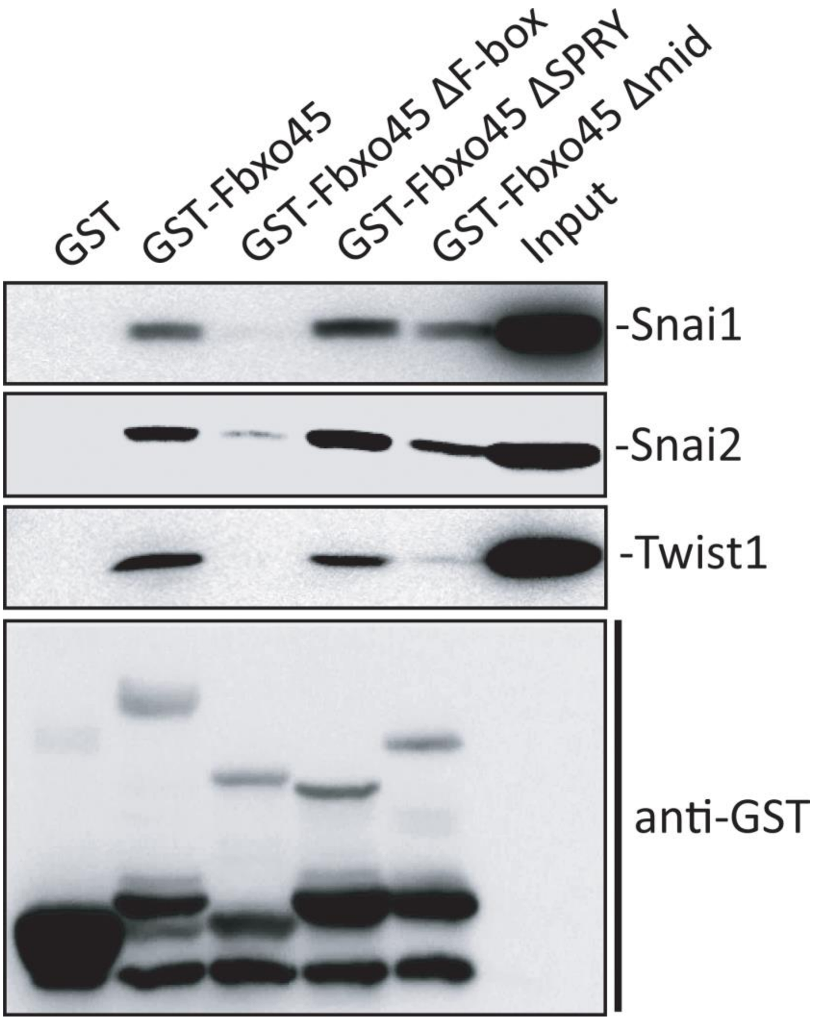

Figure S4: GST-tagged forms of Fbxo45, Fbxo45ΔF-box, Fbxo45Δmid, or Fbxo45ΔSPRY were pulled down and purified using Glutathione Sepharose 4B to precipitate the whole lysate of HEK293T cells expressed with exogenous Flag-tagged Snai1, Snai2 and Twist1 for SDS-PAGE and western-blot analysis by using the protein antibodies of Flag M2. GST-tagged WT or truncated forms of Fbxo45 were detected by the mouse GST antibody.

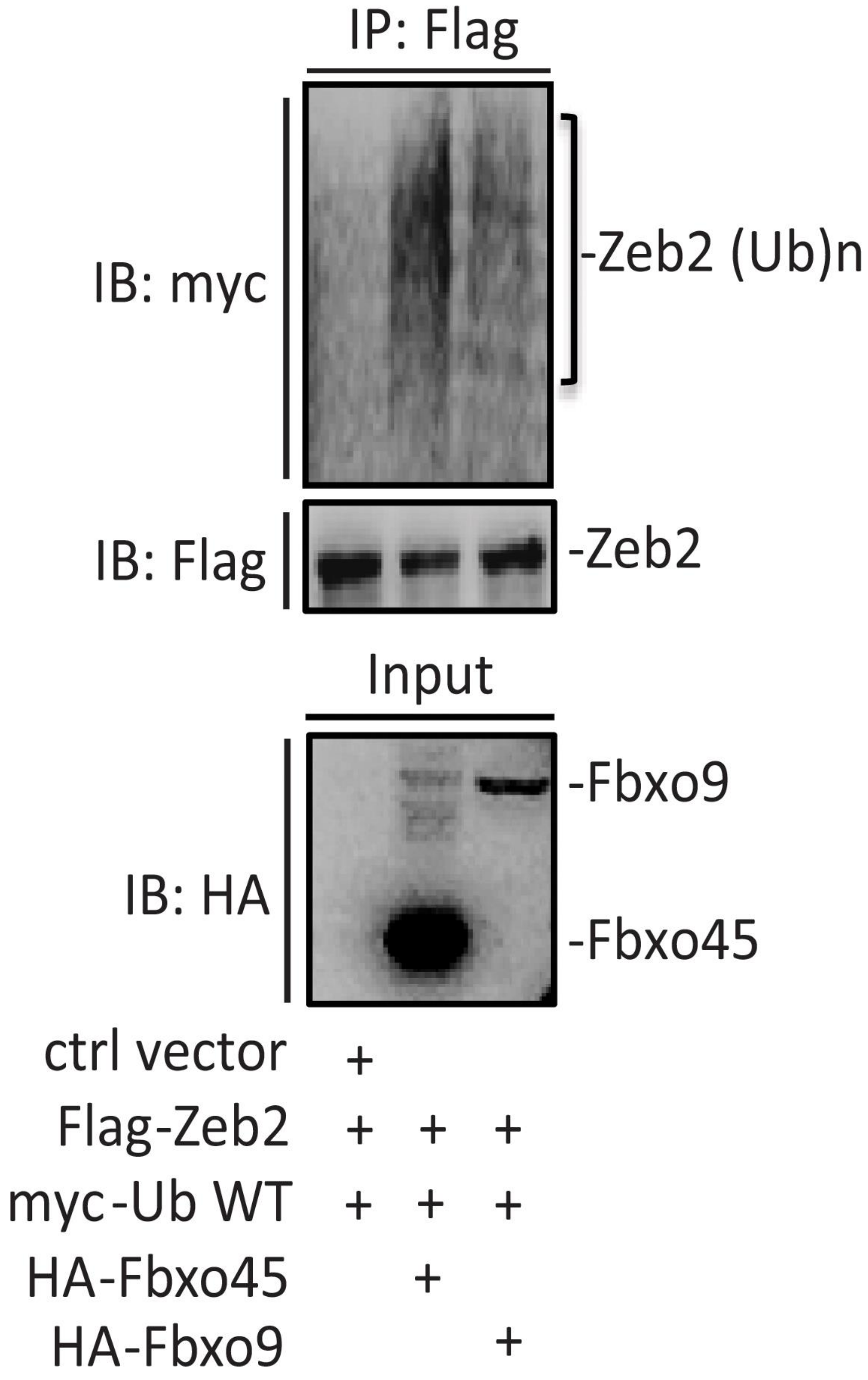

Figure S5: Lysates from 293T cells transfected myc-Ub WT with 3×Flag-Zeb2, and HA-Fbxo45 or HA-Fbxo9 were immunoprecipitated with anti-Flag M2 antibody, and then immunoblotted for ubiquination analysis.

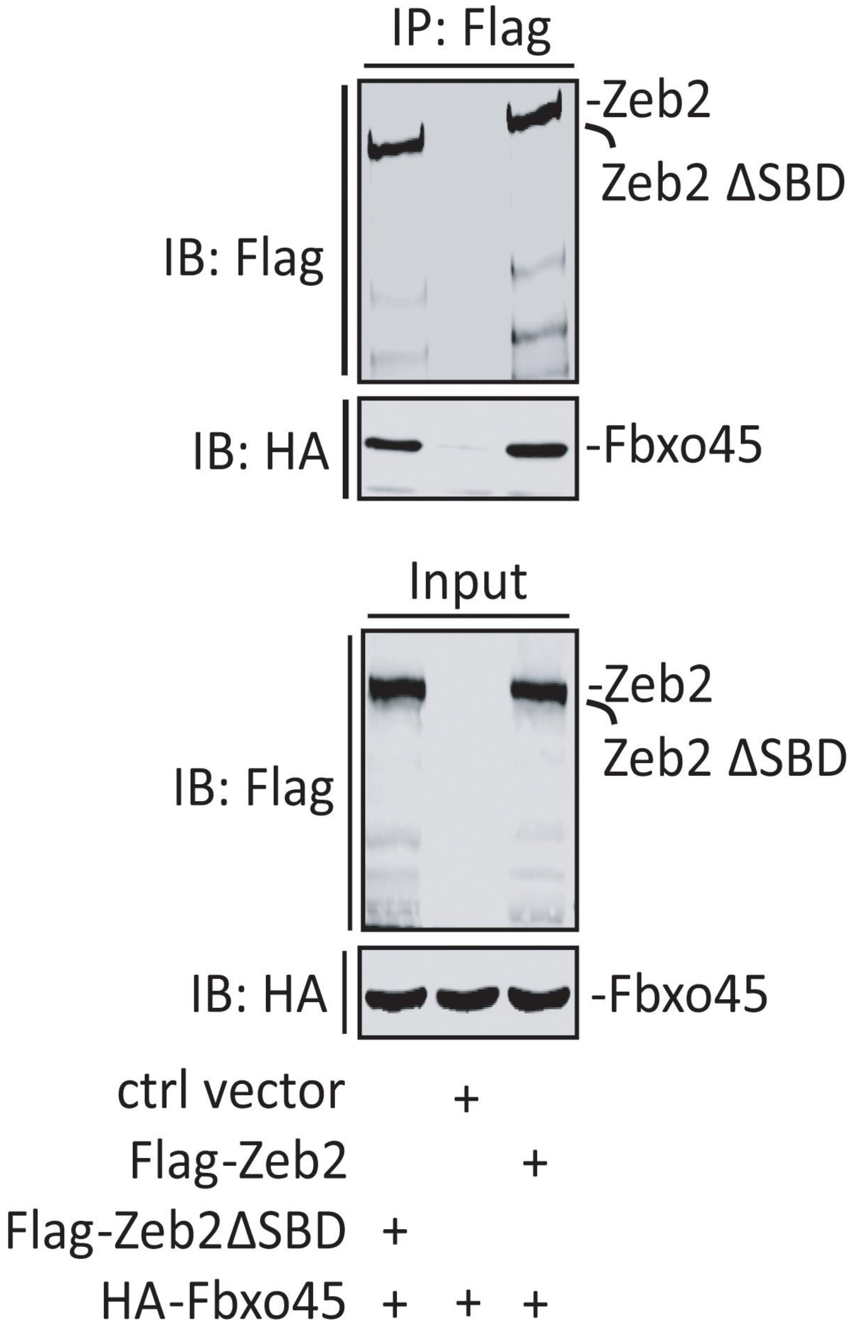

Figure S6: SBD domain of Zeb2 is essential for its ubiquitination. S6. Full length or SBD-truncated forms of Zeb2 protein with 3×Flag-tag were immunoprecipitated using Flag M2 antibody from HEK293T whole cell extraction and binding protein Fbxo45 tagged with HA was detected through western-blot analysis.

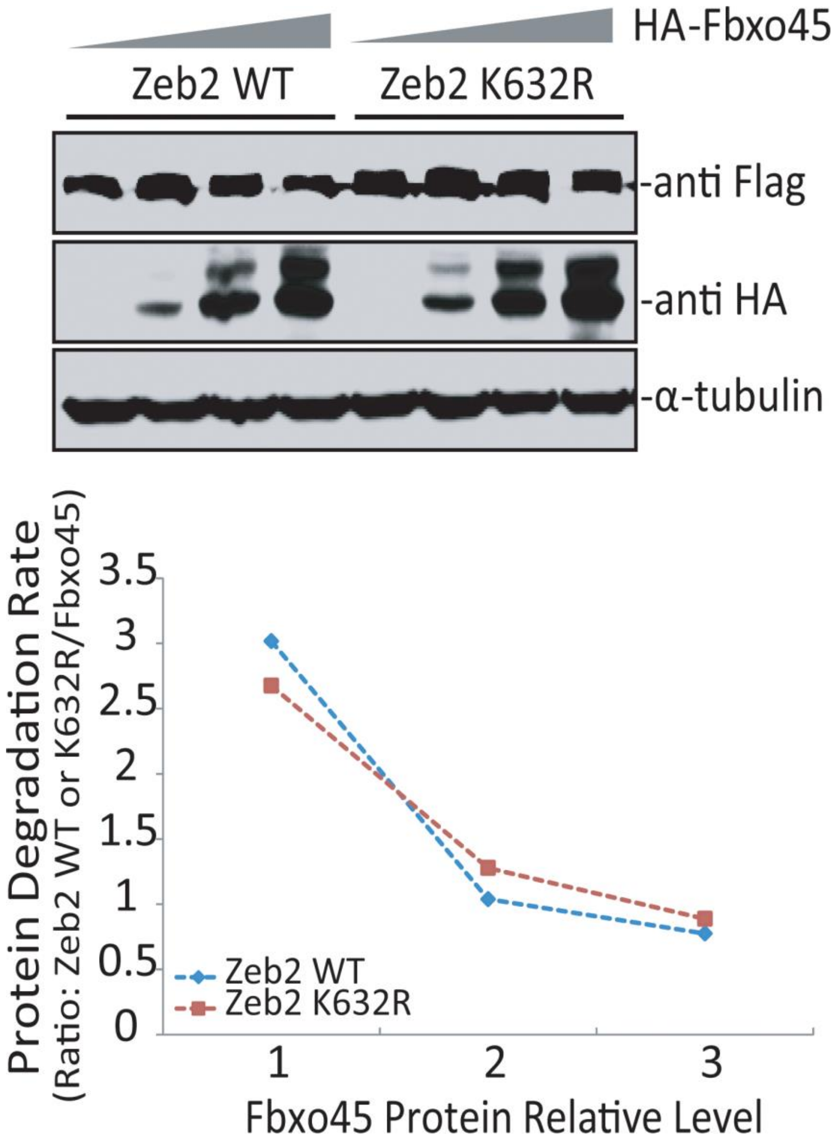

Figure S7: Wide type or K632R mutant Zeb2 protein with 3×Flag-tag were co-expressed with gradient HA-tagged Fbxo45 in HEK293T cells and the lysates were analyzed by western-blot (Upper). Image J software was used to detect the grey intensity of protein bands for the production of line chart (Under).

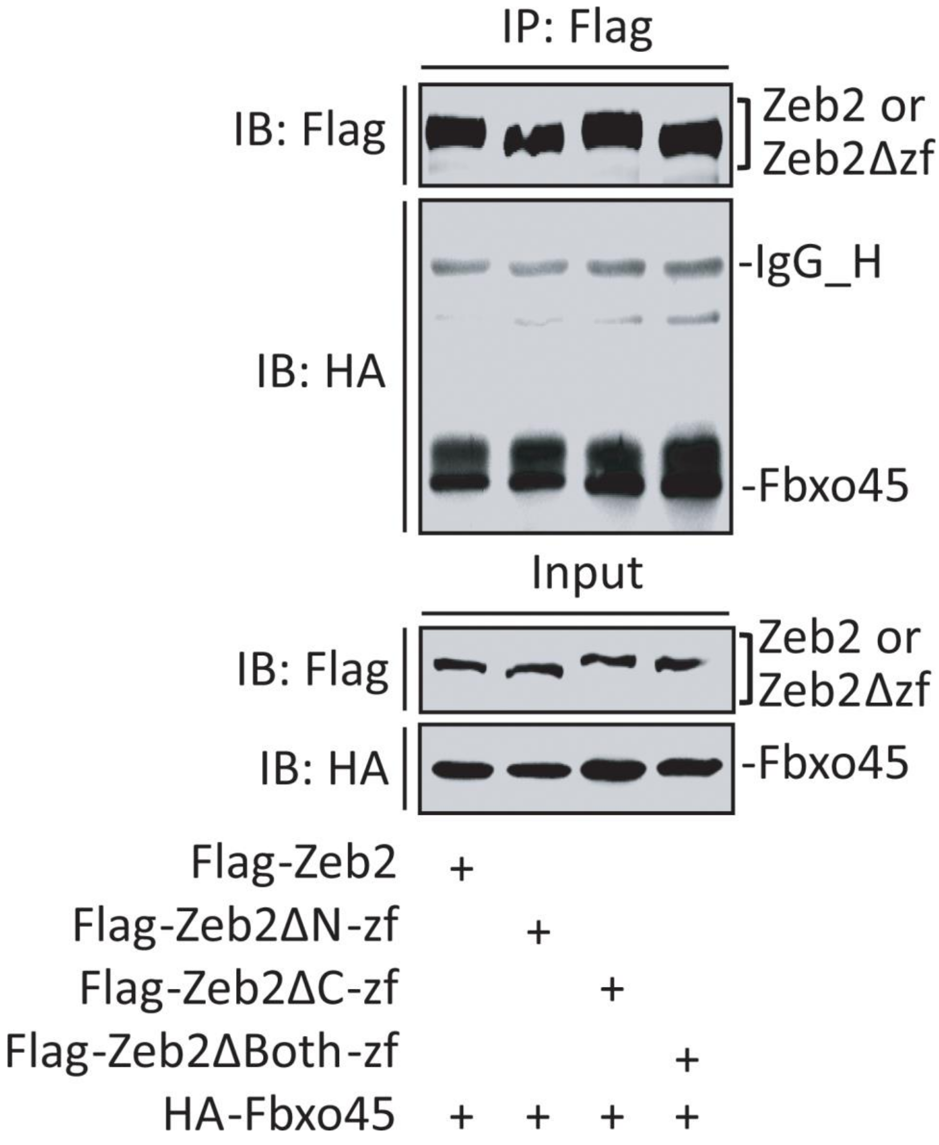

Figure S8: SBD domain of Zeb2 is essential for its Ubiquitination. Full length or zinc finger-truncated forms of Zeb2 protein with 3×Flag-tag were immunoprecipitated using Flag M2 antibody from HEK293T whole cell extraction and binding protein Fbxo45 tagged with HA was detected through western-blot analysis.

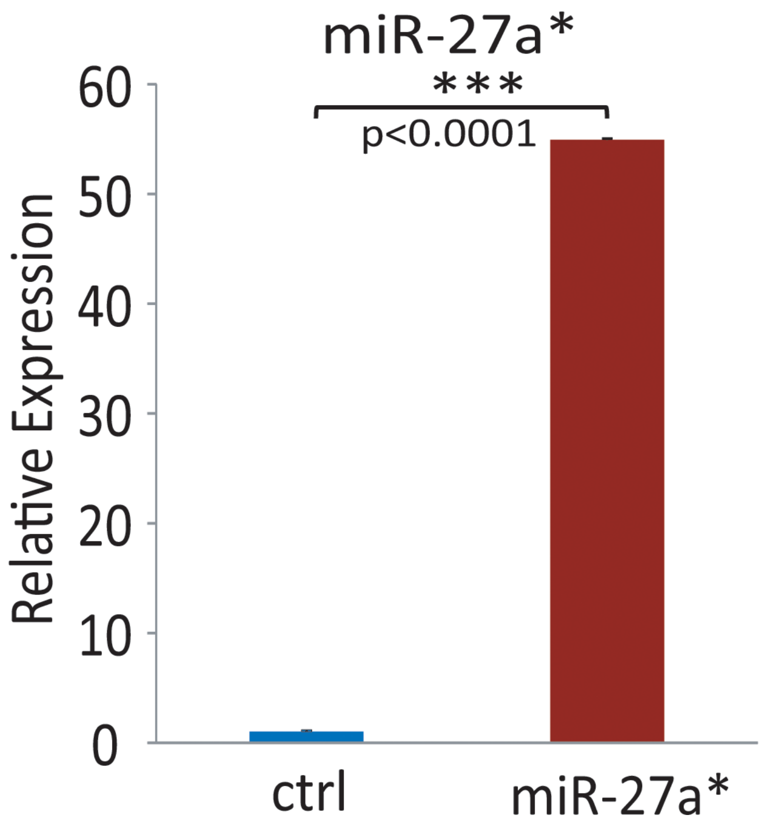

Figure S9: Fbxo45 is a direct target of miR-27a* that mediates EMT processes. The mRNA level of miR-27a* in P69 cell line infected with recombinant lentivirus expressing mature miR-27a* were confirmed by qRT-PCR method. Cell line infected with lentivirus packaged from pGreenPuro empty vector was used as the negative control (ctrl).

Table.S1: List of DNA or RNA oligonucleotides

Names	Oligonucleotides (5'- 3')
**Zeb2 F:**	AATGCTAGCGCCACCATGGGAAAGCAGCCGATCATGGCGGATGGCC
**Zeb2 R:**	TCCGCGATCGCAGATCCTTTTACATGCCATCTTCCATATTGTC
**3xFlag-Zeb2 F:**	ATCGATTACAAGGATGACGATGACAAGCTCGAGAAGCAGCCGATCATGGCGGATGGCCC
**3xFlag-Zeb2 R:**	ATCATGATCTTTATAATCACCGTCATGGTCTTTGTAGTCCATGGTGGCGCTAGCTCTAGAATCTTC
**Zeb2 ZSH F:**	GATTACAAGGATGACGACGATAAGTAAAAG
**Zeb2 ZSH R:**	TTTTCGTTGTTCAAACCATTCCTTCAC
**Zeb2 CZ F:**	GTCTACCAGTACTCAAATTCCAGGTCC
**Zeb2 CZ R:**	CTTGTCATCGTCATCCTTGTAATCGATATC
**Zeb2ΔN-Zf F:**	TCCCATTCTGGTTCCTACAGTTCGCAC

**Zeb2ΔN-Zf R:**	GTTCTCTTCATTCTTCTCGTGGCGGTAC
**Zeb2ΔC-Zf F:**	TCGGGCTCGTACTCGCAGCACATGAATC
**Zeb2ΔC-Zf R:**	ACTGCTTTTCTGGAATGTCTTGTCAC
**Zeb2 K632R F:**	AGAGCCCTCCTCTTGTCATCTGTACTTTCTG
**Zeb2 K632R R:**	ATTATCAACAAAAACTCCGGCTTTGTTGGG
**Fbxo45 F:**	AATGTCGACCGCGGCGCCGGCCCCGGGG
**Fbxo45 R:**	AGTGCGGCCGCTCATCCGTCCAAAGGTTTTCCAAGG
**Fbxo45ΔF-box F:**	AGCCTGGCAGAAGAGGCTCTGCGCACG
**Fbxo45ΔF-box R:**	GGTCGACCGAATTCGGGCCTCCATGGC
**Fbxo45ΔSPRY F:**	TGAGCGGCCGCGGGGATCCAGACATG
**Fbxo45ΔSPRY R:**	CTCACTGAAACCAATCTTGGTCCTTGC
**Fbxo45Δmid F:**	GGCCGCCATGCATGGGAAGTGTGGTGG
**Fbxo45Δmid R:**	GCGGGCGCACAGGCTCCGCCACAC
**miR-27a* F:**	GATCCGAGGGCTTAGCTGCTTGTGAGCACTTCCTGTCAGATGCTCACAACGTGCTAAGCCCTCTTTTTG
**miR-27a* R:**	AATTCAAAAAGAGGGCTTAGCACGTTGTGAGCATCTGACAGGAAGTGCTCACAAGCAGCTAAGCCCTCG
**zipmiR-27a* F:**	GATCCGAGGGCTTAGGACCTTGTGAGCACTTCCTGTCAGATGCTCACAAGCAGCTAAGCCCTCTTTTTG
**zipmiR-27a* R:**	AATTCAAAAAGAGGGCTTAGCTGCTTGTGAGCATCTGACAGGAAGTGCTCACAAGGTCCTAAGCCCTCG
**siFbxo45 1#**	GGCUUUACUUUACAUCGAATT (sense)<br>UUCGAUGUAAAGUAAAGCCTT (antisense)
**siFbxo45 2#**	GGACAAUAAUCUACUACAUTT (sense)<br>AUGUAGUAGAUUAUUGUCCTT (antisense)
**siPAM 1#**	CCCGAGAUCUUGGGAAUAATT (sense)<br>UUAUUCCCAAGAUCUCGGGTT (antisense)
**siPAM 2#**	GUGGACAUUUGGUGUAAAUTT (sense)<br>AUUUACACCAAAUGUCCACTT (antisense)
**siRNA NC:**	UUCUCCGAACGUGUCACGUTT (sense)<br>ACGUGACACGUUCGGAGAATT (antisense)
**Fbxo45 3’-UTR F:**	TATCTCGAGGAACTCCTGACCTTGTGATCCAC
**Fbxo45 3’UTR R:**	TTTGCGGCCGCACCAGTATTTTTGAGTAGAGGGGAG
**Fbxo45 3’UTR^{mut} F:**	ATTCGGGAATAGGCAGGTGCTCACATATTTG
**Fbxo45 3’UTR^{mut} R:**	CATGTTGTGGCACAAAGATGAGTC
**miR-27a* mimics:**	AGGGCUUAGCUGCUUGUGAGCA (sense)<br>CUCACAAGCAGCAGCUAAGCCCUUU (antisense)
**miR-27a* Rev:**	GTCGTATCCAGTGCAGGGTCCGAGGTATTCGCACTGGATACGACTGCTCA
**miR-27a* Q1:**	GCCTGAGGGCTTAGCTGCTTG

**miR-27a* Q2:**	GTGCAGGGTCCGAGGT
**Fbxo45 Q1:**	CAGTGCAACAACGCACCAAAATATC
**Fbxo45 Q2:**	CTCTAAAAGCAACCCCCAGGAACTC
**GAPDH Q1:**	ATGAGGTCCACCACCCTGTT
**GAPDH Q2:**	CTCAAGGGCATCCTGGGCTA
**U6 F:**	CGCTTCGGCAGCACATATAC
**U6 R:**	AGGGGCCATGCTAATCTTCT
